# The differentiated airway epithelium infected by influenza viruses maintains the barrier function despite a dramatic loss of ciliated cells

**DOI:** 10.1038/srep39668

**Published:** 2016-12-22

**Authors:** Nai-Huei Wu, Wei Yang, Andreas Beineke, Ronald Dijkman, Mikhail Matrosovich, Wolfgang Baumgärtner, Volker Thiel, Peter Valentin-Weigand, Fandan Meng, Georg Herrler

**Affiliations:** 1 Institute of Virology, University of Veterinary Medicine Hannover, Hannover, Germany; 2 Institute of Pathology, University of Veterinary Medicine Hannover, Hannover, Germany; 3 Federal Department of Home Affairs, Institute of Virology and Immunology, Bern, Switzerland; 4 Department of Infectious diseases and Pathobiology, Vetsuisse Faculty, University of Bern, Switzerland; 5 Institute of Virology, Philipps University, Marburg, Germany; 6 Institute for Microbiology, University of Veterinary Medicine Hannover, Hannover, Germany

## Abstract

Virus-host interactions in the respiratory epithelium during long term influenza virus infection are not well characterized. Therefore, we developed an air-liquid interface culture system for differentiated porcine respiratory epithelial cells to study the effect of virus-induced cellular damage. In our well-differentiated cells, α2,6-linked sialic acid is predominantly expressed on the apical surface and the basal cells mainly express α2,3-linked sialic acid. During the whole infection period, release of infectious virus was maintained at a high titre for more than seven days. The infected epithelial cells were subject to apoptosis resulting in the loss of ciliated cells together with a thinner thickness. Nevertheless, the airway epithelium maintained trans-epithelial electrical resistance and retained its barrier function. The loss of ciliated cells was compensated by the cells which contained the KRT5 basal cell marker but were not yet differentiated into ciliated cells. These specialized cells showed an increase of α2,3-linked sialic acid on the apical surface. In sum, our results help to explain the localized infection of the airway epithelium by influenza viruses. The impairment of mucociliary clearance in the epithelial cells provides an explanation why prior viral infection renders the host more susceptible to secondary co-infection by another pathogen.

The airway epithelium is the primary barrier to infection by respiratory pathogens. Viruses have found different ways to get across the epithelial barrier, such as transcytosis^1^ or via infected immune cells^2^,^3^. The most straightforward strategy, however, is the infection of the epithelial cells. For this purpose, the pathogens have to overcome the mucociliary clearance system made up from mucins released by mucus-producing cells. Foreign material entrapped by the mucus is transported out of the respiratory tract by the ciliated cells^4^,^5^.

Influenza A viruses (IAV) are rather efficient in overcoming the defence mechanisms of the host using their two surface glycoproteins, hemagglutinin (HA) and neuraminidase (NA), which have sialic acid binding and neuraminidase activities^6^,^7^,^8^. Infection of the airway epithelial cells is initiated by the binding of the haemagglutinin to cell surface glycoconjugates. Human and swine IAV (swIAV) preferentially bind to α2,6-linked sialic acid, whereas most avian IAV have a preference for α2,3-linked sialic acid^9^. To enter host cells by fusion of the viral and the cellular membrane, the haemagglutinins of mammalian IAV are activated in the respiratory tract by proteases like TMPRSS2 and HAT^10^.

Infections by human and swIAV usually remain restricted to the respiratory tract. The distribution of activating proteases may in part explain the localized infection induced by these viruses^11^. However, the interactions between IAV and airway epithelial cells that result in cellular damage on the one side and in the recovery of the respiratory epithelium on the other side are not well characterized.

The primary target cells of mammalian IAV are the differentiated airway epithelial cells. Here we established a swine air-liquid interface (ALI) culture system for long term infection studies. The well-differentiated primary porcine tracheal epithelial cells (PTEC) and porcine bronchial epithelial cells (PBEC) provide a suitable *in vitro* model to mimic *in vivo* conditions of the airway epithelium. We used these swine ALI cultures to monitor the changes in the respiratory epithelium associated with an IAV infection.

## Results

### An air-liquid interface culture system for differentiated porcine airway epithelial cells

**Figure 1:**
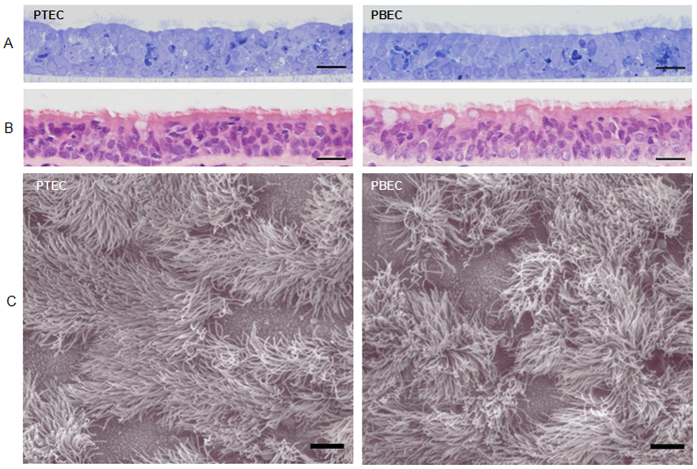
Morphological examination of porcine well-differentiated airway epithelial cell cultures. (**A**) PTEC and PBEC cultures were grown under ALI conditions for more than 4 weeks. The semi-thin sections followed by toluidine blue staining were performed. (**B**) Epithelia from porcine trachea and primary bronchus were collected, followed by histological sectioning and H&E staining for the morphological comparison. The histological examination was evaluated by light microscopy and the representative histological sections (40x magnification) are shown. (**C**) The micrograph of the scanning electron microscopy illustrates the apical surface of PTEC and PBEC. The ciliated epithelial cells are the predominant cell type. Scale bars, 20 μm (**A,B**), 5 μm (**C**).

**Figure 2:**
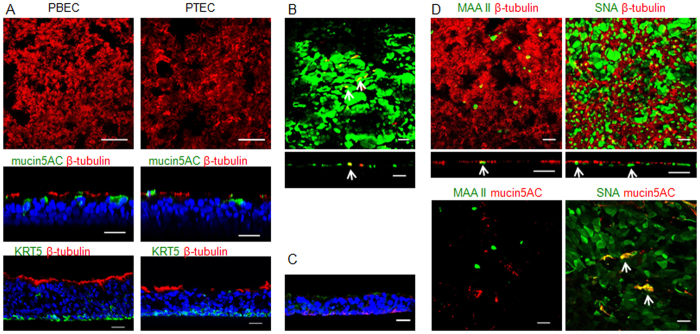
Characterization of porcine well-differentiated airway epithelial cell cultures. PTEC and PBEC were cultured under ALI conditions for at least 4 weeks and analyzed by immunofluorescence. (**A**) Immunofluorescent staining of whole-filter cultures (top and middle panels) or cryosections (lower panels) of PTEC and PBEC. The cilia are stained in red by using anti-β-tubulin antibody (top panels in horizontal sections and middle panels in vertical sections). More than half of the PTEC and PBEC surface was covered by cilia. The positive staining of mucus (green, mucin 5AC monoclonal antibody, middle panels in vertical sections) indicated the presence of mucus-producing cells. The basal cells were stained by antibody against cytokeratin 5 (KRT 5, green, lower panels) and were located above the filter support. (**B**) Detection of sialic acid on the apical surface of wdPBEC. Antibodies against SNA (green) and MAA II (red) were used to recognize α2,6- and α2,3-linked sialic acids, respectively. The images are shown in horizontal (top) or vertical (lower) sections. (**C**) Detection of sialic acids on basal cells in wdPBEC. Cryosections of PBEC cultures were stained by SNA (green) and MAA II (red). (**D**) The distribution of sialic acids in wdPBEC. PBEC stained for SNA or MAA II (green) were co-stained for the presence of cilia or mucus (red). The images are shown in vertical (middle panels) or horizontal (others) sections. The pseudo-colour was applied in red (mucin 5AC) and green (MAA II) by using LAS AF Lite software for image comparison (lower left panel). The arrows show co-localization. Scale bars, 50 μm (**A**, top), 25 μm (others).

To study the IAV infection in differentiated airway epithelial cells, we established an ALI culture system derived from the porcine airway. Primary PTEC and PBEC were isolated from the tracheae and bronchi, respectively, of swine that were shown by multiplex PCR to be negative for porcine respiratory tract pathogens. PTEC and PBEC were cultured under ALI conditions for four weeks. Histological staining of semi-thin sections indicated that both cultures showed the characteristic appearance of a pseudostratified ciliated columnar epithelium (Fig. 1A), similar to that obtained by H&E staining of tissue derived from the primary bronchus and trachea of swine (Fig. 1B). Examination by scanning electron microscopy revealed that the majority of cells contained cilia (Fig. 1C). Furthermore, PTEC and PBEC were shown by fluorescent staining to contain ciliated, mucus-producing cells and basal cells (Fig. 2A). These data indicate that the airway epithelial cells were well-differentiated. There were no major differences in the results obtained with PTEC and PBEC. Therefore, in the following part only results obtained with PBEC are shown.

### Sialic acid distribution on PBEC

The sialic acid distribution on well-differentiated PBEC (wdPBEC) cultures was determined by lectin staining. Both α2,3-linked (red) and α2,6-linked sialic acids (green) were expressed on the apical surface of PBEC cultures but the α2,6-linkage type was predominant (Fig. 2B). Co-localization of *Maackia amurensis* agglutinin II (MAA II) and *Sambucus nigra* agglutinin (SNA) signals (Fig. 2B, arrows) indicates that some cells expressed sialic acids in both linkage types. When lectins were applied from the basal side, wdPBEC were primarily stained by MAA II and hardly by SNA (Fig. 2C). Strong MAA II staining is found in the same area that is stained by the basal cell marker (compare 2A and 2C) indicating that basal cells mainly contain α2,3-linked sialic acids. The distribution of sialic acids in wdPBEC was analyzed also by co-staining of sialic acids and cilia or mucus markers. Both α2,3 and α2,6-linked sialic acids (green) were detected on ciliated cells (red), while non-ciliated cells predominantly contained α2,6-linked sialic acid (Fig. 2D). Mucin/mucus-producing cells (red) were found to have α2,6-linked but not α2,3-linked sialic acids (Fig. 2D).

### Replication kinetics of human IAV

**Figure 3:**
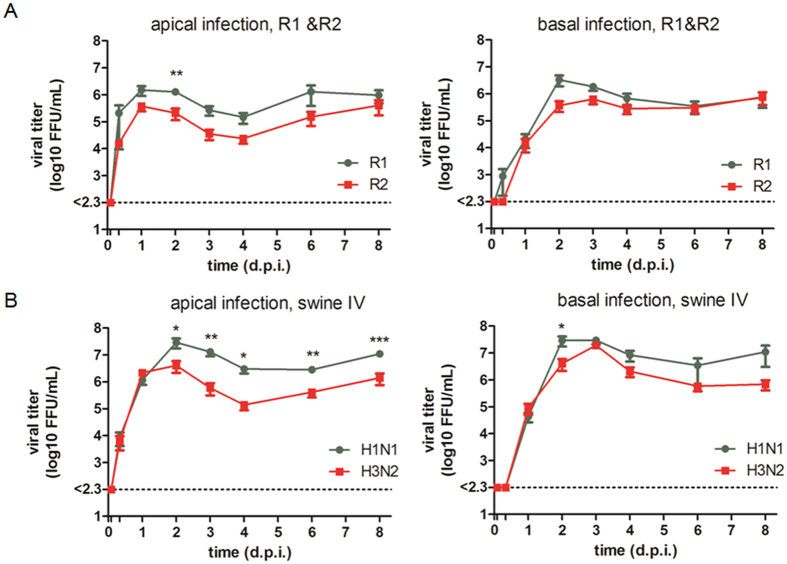
Replication of influenza A viruses in porcine well-differentiated airway epithelial cell cultures. (**A**) Replication kinetics of R1 and R2 viruses in wdPBEC. (**B**) Replication kinetics of swIAV in wdPBEC. WdPBEC were inoculated with IAV from apical (left panels) or basolateral (right panels) sides at an MOI of 0.25. Viruses released from the apical side were harvested at different time points and titrated by focus-forming assay in MDCK cells. The results were shown as means ± SEM of nine PBECs from three independent donors (swIAV) or six PBECs from two donors (R1 and R2). Each sample was processed with two technical replicates. It should be noted that some error bars are too small to be printed. Statistical analysis was performed with two-tailed unpaired Student’s *t*-test (***P < 0.001, **P < 0.01, *P < 0.05).

We used two recombinant human IAV, R1 and R2, to analyze the course of infection in ALI cultures. R1 is derived from the pandemic strain A/Hong Kong/1/68 (H3N2) and has a binding preference to α2,6-linked sialic acids. R2 differs from R1 by two mutations in the haemagglutinin and prefers binding to α2,3-linked sialic acid^12^. WdPBEC cultures on filter supports were inoculated by R1 or R2 from the apical surface at an MOI of 0.25. At all time points analysed, the titre of R1 virus released from the apical side of wdPBEC was somewhat higher than that of R2 (Fig. 3A). This result is consistent with the abundant presence of the α2,6- linkage type on the apical surface, but it also shows that R2 is able to replicate to substantial titres despite the low amount of α2,3-linked sialic acid detected by MAA staining (Fig. 2B). When IAV was applied from the basal side of wdPBEC, there was no significant difference in the replication kinetics irrespective of the binding preference for α2,3- or α2,6-linked sialic acids (Fig. 3A).

### Replication kinetics of swIAV

**Figure 4:**
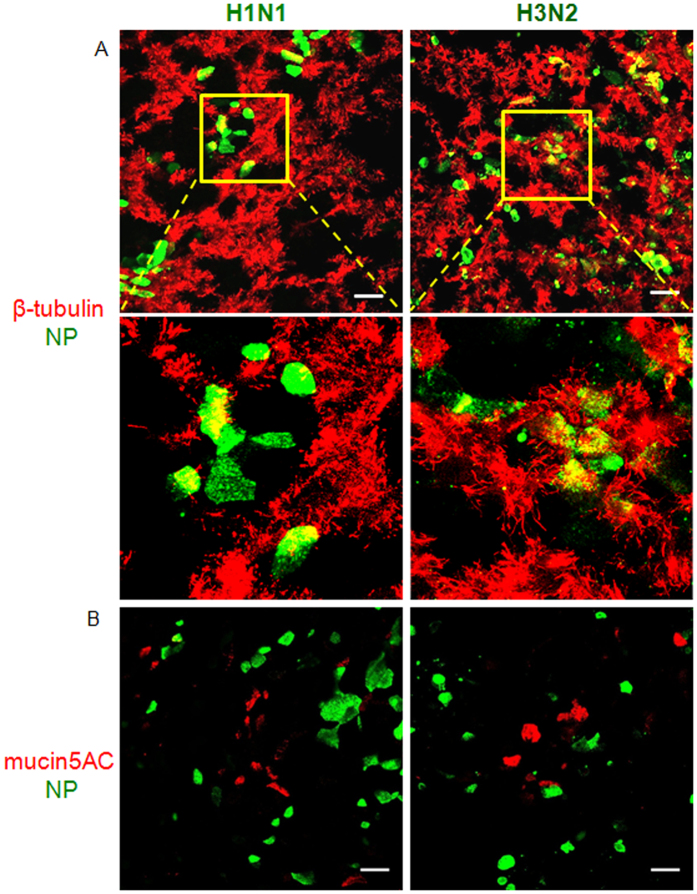
Differences in tropism of swIAV to distinct types of differentiated airway cells. WdPBECs were infected with swIAV H1N1 or H3N2 from the apical surface at an MOI of 0.25 and fixed at 1 dpi, followed by immunofluorescent staining to detect viral nucleoprotein (green), cilia (**A**, red) and mucus (**B**, red). Magnifications of squared areas are presented in the lower panels of A. Scale bars, 25 μm.

To analyze the replication of swIAV, wdPBEC cultures were infected by either of two swIAV strains (H1N1 or H3N2 subtype) from the apical side. Starting from 2 dpi, the titre of released swIAV H1N1 was significantly higher than that of swIAV H3N2 (Fig. 3B). As shown in Fig. 4, swIAV antigen (green) was associated with β-tubulin-positive cells (red) and also with β-tubulin-negative cells (Fig. 4A), but not with mucin5AC-positive cells (red) at 1 dpi (Fig. 4B). These results demonstrate that both swIAV strains preferentially infect ciliated cells and some non-ciliated cells different from mucus-producing cells.

### SwIAV-induced apoptosis and damage of the mucociliary clearance system

**Figure 5:**
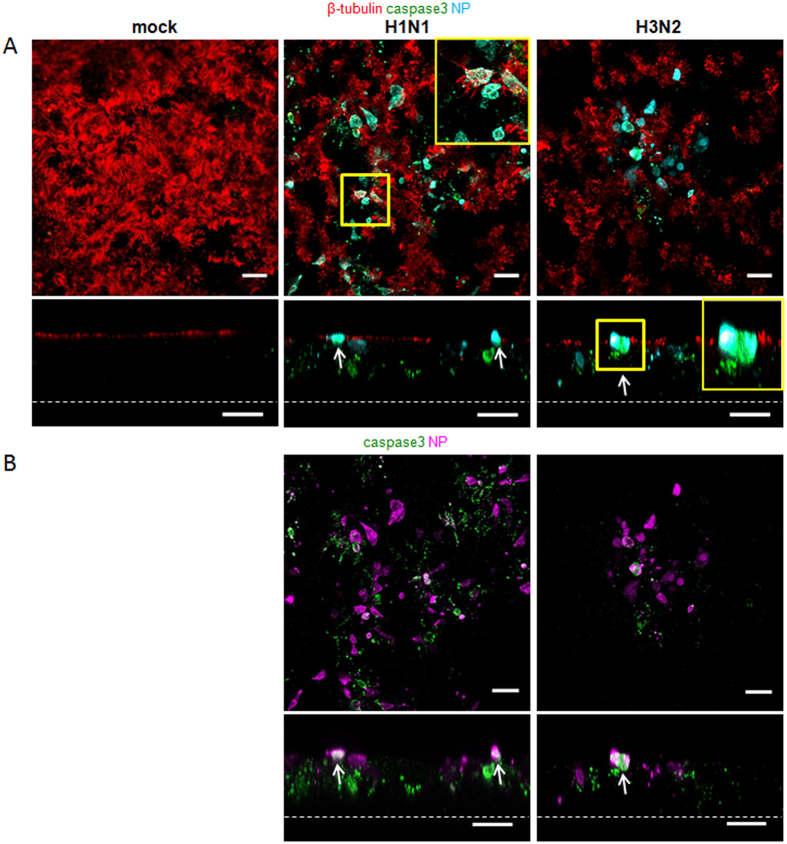
Immunofluorescent staining of apoptotic cells in PBEC at 2 days post infection. WdPBECs were infected by swIAV from the apical surface at an MOI of 0.25 and fixed at 2 dpi. The cilia (red) and the viral nucleoprotein (A, cyan or B, magenta) were stained. The apoptotic cells were detected by visualizing cleaved caspase-3 (green). Confocal images are shown in horizontal (top panels) or vertical (lower panels) sections. Magnifications of squared areas are presented on the top-right corner (**A**). The pseudo-colour was applied in magenta (viral nucleoprotein) by using LAS AF Lite software for image comparison (**B**). The arrows show co-localization, and the dashed lines indicate the location of the supporting membrane. Scale bars: 25 μm.

To analyze the detrimental effect of IAV infection on airway epithelial cells, we performed immunofluorescent staining to detect apoptotic signals and monitor the morphological changes. At 2 dpi, cells positive for activated caspase-3 (green) were present in swIAV-infected wdPBEC (Fig. 5). Moreover, co-localization of cleaved caspase-3, β-tubulin and viral nucleoprotein (NP) is shown in Fig. 5A (arrows), and co-localization of caspase-3 and NP in Fig. 5B indicating that swIAV infection induces apoptosis in ciliated cells.

**Figure 6:**
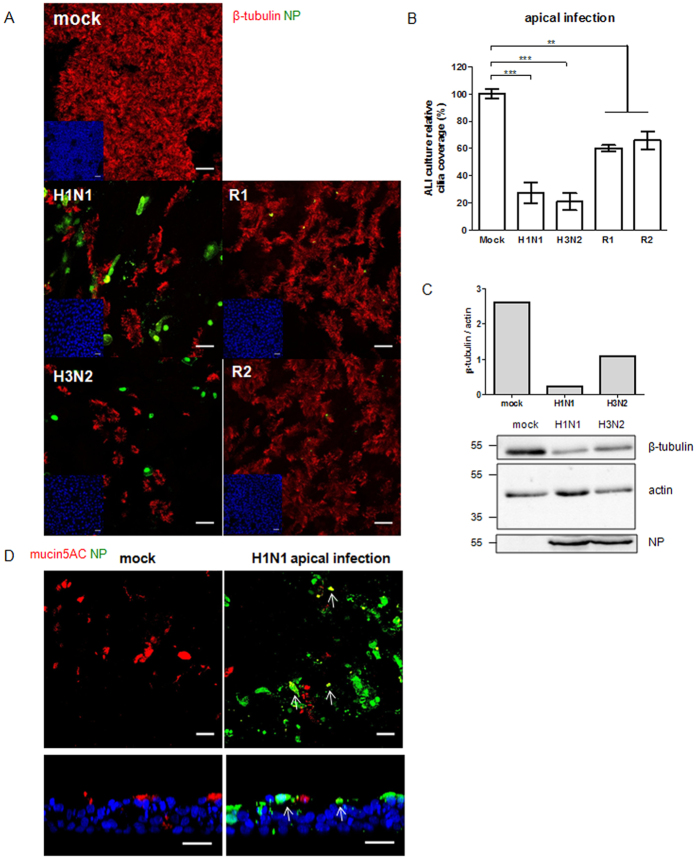
Immunofluorescent staining of porcine well-differentiated airway epithelial cells at 8 days post infection. WdPBECs were inoculated with IAV from the apical side at an MOI of 0.25 and fixed at 8 dpi. (**A**) PBEC cultures were stained for viral nucleoprotein (green) and cilia (red). (**B**) Quantification of the ciliated area at 8 dpi. Results are shown as percentages (means ± SEM) compared to mock-infected cultures. For each infection, six PBECs from three independent donors were measured, and three fields per culture were evaluated as technical replicates. (**C**) Western blot analysis of β-tubulin expression level in PBECs after swIAV infection. The relative expression level of β-tubulin was normalized to actin expression. The viral NP could be detected in the infected culture. (**D**) Immunofluorescent staining for viral nucleoprotein (green) and mucin (red). The nuclei were stained by DAPI (blue) (**A and D**). The arrows show co-localization. Scale bars, 25 μm.

**Figure 7:**
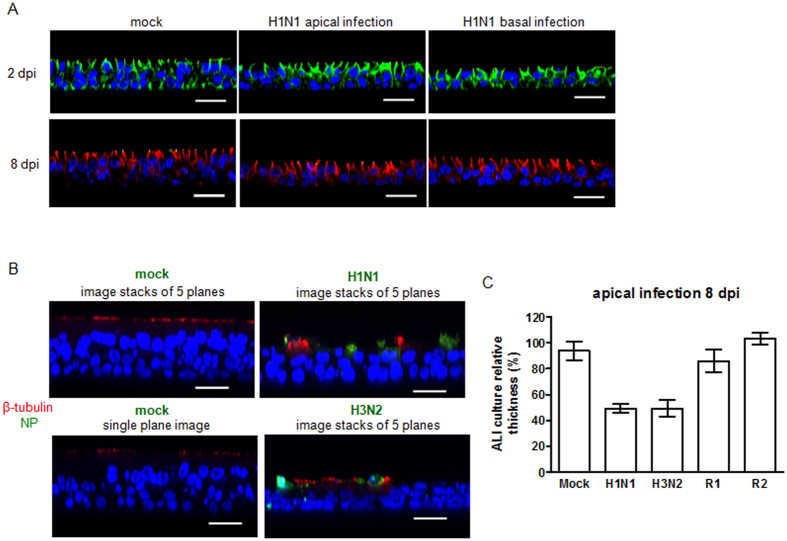
Decreased thickness of porcine well-differentiated airway epithelial cell cultures after IAV infection. WdPBECs were inoculated by IAV from the apical (middle panels of A; B and C) or basolateral side (right panels of A) at an MOI of 0.25. (**A**) wdPBEC were inoculated with swIAV H1N1 and fixed at 2 or 8 dpi, followed by immunofluorescent staining with antibody against the adherens junction protein β-catenin (green at 2 dpi; red at 8 dpi). Confocal images are shown in vertical sections. (**B**) WdPBECs were fixed at 8 dpi and stained for viral nucleoprotein (green) and cilia (red) (vertical sections). To measure the thickness accurately, the vertical image stacks of 5 planes (distance of 1.0 μm per plane) were merged. It should be noted that the epithelium forms a pseudostratified layer in the single plane image. The nuclei were stained by DAPI (blue) (A&B). (**C**) Quantification of wdPBEC thickness at 8 dpi. Results are shown as percentages (means ± SEM) compared to mock-infected ALI cultures. For each infection, numbers of six PBECs from three independent donors were measured. Additionally, three fields per culture were evaluated as technical replicates. Scale bars, 25 μm.

To get more information on the long term influence caused by IAV infection, we performed quantitative analyses of the levels of ciliated cells and epithelial thickness. At 8 dpi, a loss of cilia was observed in all virus-infected wdPBEC compared to the mock-infected cultures. The swIAV-infected wdPBEC lost more cilia compared to R1- and R2-infected samples as determined from the area covered by cilia (Fig. 6A and 6B). DAPI-staining of nuclei (Fig. 6A, insets) indicated that the virus-infected PBEC were still present as a confluent cell layer. Moreover, the loss of cilia in swIAV-infected wdPBEC was confirmed by Western blot analysis (Fig. 6C). Despite of the failure to detect infection of mucus-producing cells at 1 dpi (Fig. 4B), co-localization of mucin and NP was observed at 8 dpi (Fig. 6D, arrow). Furthermore, the thickness of swIAV-infected cultures was reduced by about 50% at 8 dpi (Fig. 7); in R1- or R2-infected PBEC, the epithelial thickness was not significantly reduced (Fig. 7C).

### Maintenance of the barrier function of virus-infected PBEC

**Figure 8:**
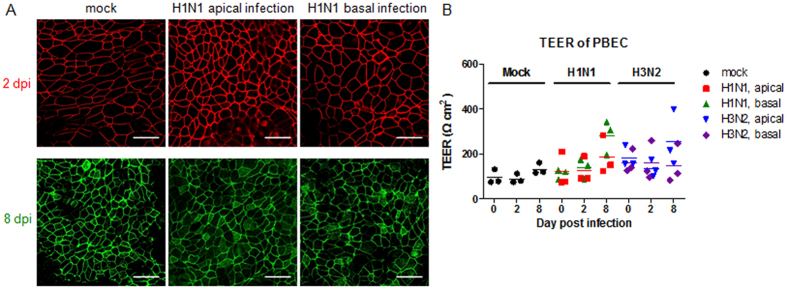
Porcine well-differentiated airway epithelial cell cultures preserve tight junctions after swIAV infection. (**A**) WdPBECs were inoculated with swIAV H1N1 from the apical (middle panels) or basolateral (right panels) side at an MOI of 0.25. ALI cultures were fixed at 2 dpi (top panels) and 8 dpi (lower panels), followed by staining with anti-ZO-1 antibody (red in top panels; green in lower panels) to detect tight junction. Scale bars, 25 μm. (**B**) WdPBECs were inoculated by swIAV from the apical or basolateral side. The trans-epithelial electrical resistance (TEER) values of mock-infected and swIAV-infected PBEC were determined at the indicated time points. The results are shown as three PBECs from three independent donors. Each sample was performed with 3 technical replicates.

In order to determine whether or not the barrier function of PBEC is affected by the virus infection, we analyzed the cell–cell junctions and the transepithelial electrical resistance (TEER) values. The patterns of tight junctions (ZO-1, Fig. 8A) and adherens junctions (β-catenin, Fig. 7A) between individual cells were observed both at 2 and 8 dpi. During the whole infection period analyzed, the TEER values of wdPBEC (Fig. 8B) were not decreased after swIAV infection up to 8 dpi. These results indicate that the tight junctions were maintained in wdPBEC infected by swIAV.

**Figure 9:**
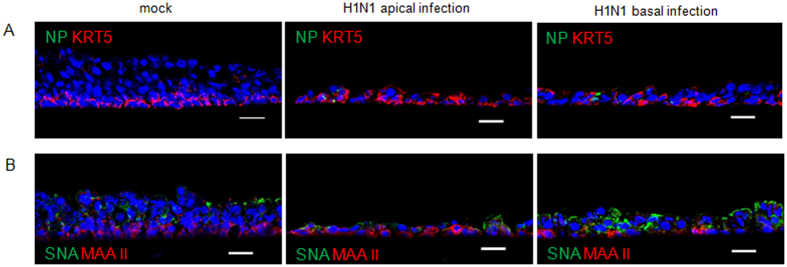
Sialic acid expression on PBEC after swIAV infection. WdPBECs were inoculated by swIAV H1N1 from the apical (middle panels) or basolateral (right panels) side at an MOI of 0.25. Cryosections were prepared at 8 dpi. (**A**) Immunofluorescent staining for KRT 5 (red, basal cells) and viral nucleoprotein (green). (**B**) Immunofluorescent staining to detect α2,3- and α2,6-linked sialic acid using MAA II and SNA lectins, respectively. The nuclei were stained by DAPI (blue) (A&B). Scale bars: 25 μm.

The cells maintaining the barrier function at 8 dpi were therefore analyzed by staining the cultures for the basal cell marker KRT5 and for the presence of sialic acids. Despite the loss of cilia on the apical surface, swIAV-H1N1-infected PBEC that had survived infection were positive for KRT5 (Fig. 9A). Some of the cells were infected by swIAV as indicated by staining for NP. Furthermore, lectin staining indicated that the KRT5-postive cells contained both α2,3- or α2,6-linked sialic acids (Fig. 9B). By contrast, in mock-infected samples, MAA II staining was mainly detected in the basal portion of the cell layer whereas SNA preferentially stained the apical portion. These results are consistent with the conclusion that the cells maintained at 8 dpi are derived from basal cells that are in the process of differentiating into specialized cells.

## Discussion

The airway epithelium is equipped with the mucociliary clearance system to prevent the detrimental effect of foreign substances including infection by microorganisms. This defence mechanism is based on the mucins produced by mucus-producing cells and the ciliary activity of specialized epithelial cells that transport the mucus out of the respiratory tract^4^,^5^. To understand the infection of airway epithelial cells, it is necessary to use culture systems that comprise mucus-producing cells and ciliated cells. To this end, we and others studied IAV infection in differentiated ALI cultures of human airway epithelial cells which contain all major cell types present in human airway epithelium *in vivo*, namely, ciliated cells, secretory cells and basal cells^12^,^13^,^14^,^15^. Here we developed and employed swine ALI (swALI) cultures that can be used to analyze the infection by both human and swine IAV^16^,^17^. Compared to the human counterpart, swALI cells have the advantage that the source of cells is well-defined and reproducible as far as the age and genetic background of the animals. The growth conditions of swALI cultures were different from those described for human ALI cultures. The difficulty in establishing this system may explain why so far only few reports about differentiated respiratory epithelial cells of swine are available^16^,^17^,^18^. The novel feature in our study is that we analysed events occurring late after infection not addressed in previous analyses with swine or human ALI cultures.

By histological examination, our well differentiated PBEC and PTEC were shown to resemble a pseudostratified ciliated epithelium as it is present in the respective portions of the swine respiratory tract. We found that the apical surface of differentiated epithelial cells predominantly contains α2,6-linked sialic acid which is consistent with other reports^16^,^19^. Although α2,3-linked sialic acids have been reported to be present on subepithelial cells of swALI cultures^16^, sialic acids of swine basal cells have not been analysed before. Here, we demonstrated that the KRT5 positive basal cells contain α2,3-linked sialic acids. When the sialic acid distribution of different species is compared, the porcine airway epithelium shows a sialic acid distribution pattern similar to that of human cells indicating that the PBEC cultures are more suitable to investigate human IAV infection than murine and ferret airway epithelial cells^20^,^21^.

The ALI culture system revealed characteristics of the IAV infection that were different from those obtained with immortalized cells. The amount of infectious virus released from the apical surface remained high during the whole infection period analyzed, i.e. up to eight dpi. This long period of virus release may be related to the ALI culture conditions. As influenza virions are released from the apical side of the polarized epithelial cells, spread of infection is expected not to be as efficient as in the case of immortalized cells which are covered by medium. Interaction with mucins may further hamper the spread of infection, because the viral binding to sialic acids has to be counteracted by the viral neuraminidase^22^.

The infection by IAV was detrimental for the airway epithelial cells as indicated by the detection of apoptotic cells. Another effect of the infection was a significant reduction in the amount of cilia detected on infected airway epithelial cells. The reduction was more pronounced in swIAV-infected cultures compared to cells infected by either of the two human IAV. The difference is consistent with a more efficient infection by swIAV as indicated by the 10-fold increased amount of virus released into the supernatant. The reduced staining of β-tubulin can be explained by the loss of ciliated cells, probably due to the apoptotic effect of the infection mentioned above. This conclusion is based on the reduced thickness of the epithelial cell layer at late times of infection that was observed in parallel to the loss of cilia staining. The loss of cilia and the reduction in epithelial thickness were also observed in swIAV-infected porcine trachea tissue explants^23^. The loss of ciliated cells was clearly detected with cultures infected by swIAV. In the case of human IAV, where the foci of infected cells were not as large as those of swIAV-infected ALI cultures, the thickness of the epithelial layer was not significantly reduced. Our results are consistent with data from IAV infections in swine. Airways infected by swIAV have been reported to be lined by flattened epithelial layers^24^.

Despite the detrimental effect of the infection by IAV, the TEER of the epithelial layer and the tight junction protein expression were retained during the whole infection period. A recent study reported IAV infection interrupts the tight junction formation in an *in vitro* alveolar culture model comprising immortalized epithelial cells^25^. In our study, however, the well differentiated tracheal and bronchial epithelia retain their barrier function. The finding of epithelial cells with tight junctions indicates that the lost ciliated cells were replaced by polarized cells that were able to maintain the barrier function of the epithelium. It appears that basal cells had started to differentiate into specialized cells. This process had not yet proceeded to the appearance of ciliated cells and mucus-producing cells, or other non-ciliated cells, but the differentiation had resulted in polarized epithelial cells that were sufficient to maintain the barrier function as indicated by the TEER. The reorganization of the epithelial cells has also been observed in a report, where mice were infected by IAV *in vivo*^26^. The intermediate position of the polarized cells is shown by our lectin staining data, which may indicate that the cells are in a differentiation process. Undifferentiated human airway epithelial cells have been shown to contain α2,3-linked sialic acids and the expression of α2,6-linked sialic acids is increasing over time during the differentiation phase^27^. Whereas α2,6-linked sialic acids are abundantly present on well-differentiated cells, basal cells mainly contained α2,3-linked sialic acids. Both linkage types of sialic acids were detected in polarized cells at late times of infection indicating an increased amount of α2,3-linked sialic acid on the apical surface of the epithelium compared to the uninfected state.

Our results provide a deeper insight into IAV infection. On the one side, there is the detrimental effect that can result in disease. On the other side, the loss of well-differentiated cells is compensated by the generation of polarized cells that maintain the barrier function of the epithelial cell layer which may contribute to the restriction of the infection to the respiratory tract. Thus, the localized infection by IAV is not only a matter of the availability of proteases required for proteolytic activation of virus infectivity but is also the result of the maintenance of the barrier function of the airway epithelium.

As the cells that replace the well-differentiated cells are not yet ciliated, the infected areas of the epithelium may for a certain time not contribute to the mucociliary clearance system. This provides an explanation why prior virus infection renders the host more susceptible to the co-infection by another viral or bacterial pathogen. In this context it is interesting to note that the above mentioned increase of the α2,3-linked sialic acids on the apical surface indicates an increased abundance of receptors for avian influenza viruses. Therefore, it is intriguing to speculate that swIAV infection renders epithelial cell more susceptible to infection by avian IAV and thus facilitates co-infection and the appearance of reassortant viruses. Such events are assumed to be responsible for the generation of pandemic viruses.

In sum, we have established an ALI culture system from porcine airway cells to analyze the long term virus infection of differentiated respiratory epithelial cells. In addition, our results not only provide new insights into the infection of the airway epithelium by IAV, they also show an experimental access to questions related to the recovery from infection, e.g. the re-differentiation of the epithelium and the susceptibility to co-infections.

## Methods

### Differentiated porcine airway epithelial cell cultures

Primary porcine airway epithelial cells were isolated from pigs of a local slaughterhouse. PTEC and PBEC were obtained from swine trachea and bronchi, respectively. Primary cells were harvested as previously described^18^ and were seeded on type I collagen (Sigma)-coated flasks with bronchial epithelial cell serum-free growth medium (BEGM). The BEGM was modified from previous studies^28^,^29^ and contained the BEBM basal medium (Lonza) supplemented with the required additives. PTEC and PBEC were transferred to type IV collagen-coated Transwell^®^ polycarbonate membrane (24 well, 0.4 μm pore size, Corning Costar) at a density of 2.5 × 10^5^ cells per filter support with the air-liquid interface (ALI) medium consisting of a mixture of DMEM (Gibco) and BEBM basal medium (1:1) with additives described previously^28^. After PTEC and PBEC reached confluence, the cells were maintained under ALI conditions for at least 4 weeks at 37 °C in a humidified 5% CO_2_ atmosphere. Both cultures were validated for porcine specific respiratory tract pathogens including porcine circovirus-2, porcine reproductive and respiratory syndrome virus, porcine cytomegalovirus, porcine influenza A virus, porcine respiratory coronavirus, *Mycoplasma hyorhinis* and *Mycoplasma hyopneumoniae* by multiplex Polymerase Chain Reaction (PCR)^30^. All PTEC and PBEC used in this study were free from the above mentioned pathogens.

### Histological examination

Porcine tracheae and bronchi were obtained from a local slaughterhouse and fixed in 10% paraformaldehyde (PFA), processed routinely, embedded in paraffin, sectioned at 5 μm, and stained with hematoxylin and eosin (H&E).

### Semi-thin sections

Staining of semi-thin sections was performed as described previously^31^. Briefly, cultured cells were fixed in 5% glutaraldehyde/cacodylate buffer for 24 hours and subsequently post-fixated with 1% osmium tetroxide. Following dehydration in a graded series of ethanol, sections were embedded in epoxy resin. The 1-μm-thick semithin sections were stained with toluidine blue and evaluated by light microscopy.

### Scanning electron microscopy

Scanning electron microscopy was performed as described previously^32^. Briefly, cultured cells were prefixed in glutaraldehyde (2.5%) for 24 hours and subsequently treated with osmium tetroxide (1%) for 2 hours. Following dehydration in an ascending series of ethanol, samples were dried under critical point drying using the E 3000 device (Polaron, USA), stuck to stubs, sputter-coated and examined under a scanning electron microscope (DSM940, Zeiss, Germany).

### Influenza viruses

Two swIAV, A/sw/Bad Griesbach/IDT5604/2006 (H1N1) and A/sw/Herford/IDT5932/2007 (H3N2), and two recombinant human IAV, R1 and R2, were used in this study. They have been described in previous reports^12^,^33^,^34^.

### Measurement of trans-epithelial electrical resistance (TEER)

The TEER developed by PTEC and PBEC cultures was measured by using the Millicell^®^ ERS-2 Voltohmmeter (Millipore) according to the manufacturer’s instructions.

### Virus infection of differentiated epithelial cells

Well-differentiated PTEC and PBEC were washed five times with PBS and inoculated with IAV from the apical or basal side at an MOI (multiplicity of infection) of 0.25; the cell number per filter support was approximately 5 × 10^5^. After 2 h of incubation at 37 °C, PTEC and PBEC were rinsed with PBS twice to remove unbound viral particles and fresh ALI medium was added. Infected PTEC and PBEC were further maintained under ALI conditions at 37 °C in a 5% CO_2_. At different time points, 100 μL of DMEM were added to the apical surface and the cultures were incubated for 30 min at 37 °C. The harvests were collected at different times post virus-infection and the viruses were titrated by focus forming assay on MDCK cells. For immunofluorescence analysis, infected PTEC and PBEC were fixed with 3% PFA or used to generate cryosections, 20 μm thick^35^.

### Lectin staining

Lectin staining was performed with *Sambucus nigra* agglutinin (SNA) or *Maackia amurensis* agglutinin II (MAAII)^34^. The binding of biotinylated lectins was visualized by fluorescence microscopy using streptavidin-Cy3 (Sigma) or streptavidin-DyLight 488 (Vector laboratories).

### Immunofluorescence analysis (IFA)

Whole-filter cultures or cryosections of PTEC and PBEC were fixed with 3% PFA and permeabilized with 0.5% Triton X-100. Cells and sections were further blocked with 5% goat serum and incubated with primary antibody, followed by incubation with Alexa Fluor^®^ conjugated secondary antibody. The nuclei were stained by DAPI (4′,6-diamidino-2-phenylindole) and were embedded with ProLong^®^ Gold Mountant (Life Technologies).

The primary antibodies used in this study were as follows: anti-influenza A virus nucleoprotein (NP) antibody (AbDSeroTec), anti-mucin-5AC antibody (Acris), anti-cytokeratin 5 (KRT5) antibody (Abcam), anti-ZO-1 antibody (Life Technologies), anti-β-catenin antibody (Sigma), cleaved caspase-3 antibody (Cell Signaling) and Cy3-labeled antibody against β-tubulin (Sigma). Secondary antibodies were Alexa Fluor^®^ 488, 568 or 633 conjugated antibodies (Life Technologies). All antibodies were diluted in 1% bovine serum albumin and incubated in RT for 1 h. IFA was performed by using a Leica TCS SP5 AOBS confocal laser scanning microscope. For the processing and analyses of confocal images, LAS AF Lite software (Leica) and ImageJ/Fuji software (National Institutes of Health) were used. The image stacks with a z- or y-distance of 1.0 μm per plane were merged. The images were generated by ImageJ/Fuji software using maximum intensity projection filter. The results were repeated at least with six PBECs from three independent donors, three fields per culture were examined by confocal laser scanning microscopy.

### Virus titration

The infectivity of the viruses was evaluated by focus forming assay on MDCK cells^36^ with modifications. Infected cells were detected with an antibody directed against influenza A virus NP and a horseradish peroxidase (HRP)-conjugated anti-mouse IgG (H + L) secondary antibody (KPL). The calculated virus titre is indicated in foci-forming units per ml (FFU/ml).

### Preparation of cell lysates and Western blot analysis

At day 8 p.i., each PTEC or PBEC was washed with PBS and lysed on ice using 50 μL RIPA buffer supplemented with protease inhibitor cocktail (Thermo Scientific). Laemmli sample buffer and 0.2 M dithiothreitol (DTT) were added to the collected samples and incubated at 96 °C for 10 min. After electrophoresis in SDS-PAGE and transfer to a nitrocellulose membrane, the samples were subjected to Western blot analysis. Anti-β-tubulin antibody (mouse, Sigma), anti-actin antibody (housekeeping control, mouse, Sigma) and anti-influenza A antibody (goat, ViroStat) served as primary antibodies and anti-mouse or anti-goat horseradish peroxidase (HRP, Dako) antibodies were used as secondary antibodies. The primary antibodies were incubated at 4 °C overnight, and the secondary antibodies were applied for 1 h at 4 °C. The specific proteins were visualized by Super Signal West Dura extended duration substrate (Thermo Scientific) and quantified by software Quantity One (Bio-Rad) and ImageJ/Fuji (National Institutes of Health). The relative protein expression levels were normalized to actin.

### Statistical analyses

Data are shown as means ± SEM. All statistical analyses were done by using Prism 5 software (GraphPad Software).

## Additional Information

**How to cite this article**: Wu, N.-H. *et al*. The differentiated airway epithelium infected by influenza viruses maintains the barrier function despite a dramatic loss of ciliated cells. *Sci. Rep.* **6**, 39668; doi: 10.1038/srep39668 (2016).

**Publisher's note:** Springer Nature remains neutral with regard to jurisdictional claims in published maps and institutional affiliations.
